# Association of Playground “Playability” With Physical Activity and Energy Expenditure

**DOI:** 10.5888/pcd20.220247

**Published:** 2023-04-27

**Authors:** Jeanette Gustat, Christopher E. Anderson, Sandy J. Slater

**Affiliations:** 1 Department of Epidemiology, School of Public Health and Tropical Medicine, Tulane University, New Orleans, Louisiana; 2 Prevention Research Center, School of Public Health and Tropical Medicine, Tulane University, New Orleans, Louisiana; 3 Department of Pharmaceutical and Administrative Sciences, School of Pharmacy, Concordia University Wisconsin, Mequon, Wisconsin

## Abstract

**Introduction:**

Children’s physical activity, especially play, is important for healthy physical, social, and psychological development. Playgrounds are public spaces for children, but not all playgrounds are conducive to play and physical activity. We examined “playability,” the ability of a space to promote active play, and associations with moderate-to-vigorous physical activity (MVPA) and energy expenditure.

**Methods:**

This cross-sectional study assessed playground features with the Play Space Audit Tool; we calculated playability scores from audit data, overall and by domain (general amenities, surface, path, and play structure), from playgrounds in 70 parks in Chicago, Illinois, in 2017. We observed 2,712 individuals during the audits and used the System for Observing Play and Recreation in Communities tool to assess MVPA and energy expenditure. We used generalized estimating equation negative binomial regression to calculate incidence rate ratios for MVPA and mixed effects models to calculate energy expenditure (in kcal/kg/min) associated with playability scores.

**Results:**

General amenities and play structure scores were associated with 1.28 (95% CI, 1.08–1.52) and 1.15 (95% CI, 1.00–1.31) times as many individuals (any age) engaged in MVPA, respectively. The general amenities score was significantly associated with 0.51 (95% CI, 0.24–0.79) and 0.42 (95% CI, 0.15–0.68) higher energy expenditure in renovated playgrounds and in all playgrounds, respectively.

**Conclusion:**

Overall, general amenities and play structure scores were associated with MVPA and were robust to adjustment for weather, neighborhood socioeconomic characteristics, and crime. These playground playability indices may strengthen future evaluations of community infrastructure for children’s physical activity.

**Summary**

**What is already known on this topic?**

Playgrounds are important public facilities for children to play and be physically active, which is essential for healthy development. Greater access to playgrounds has been associated with increased physical activity.

**What is added by this report?**

Higher scores for playground amenities and play structures were associated with 28% and 15% higher levels of moderate-to-vigorous physical activity, respectively, and higher scores for playground amenities were associated with increased energy expenditure. Observed associations between amenities and structures and active play (playability) were stronger in recently renovated playgrounds.

**What are the implications for public health practice?**

Playground features are important for promoting active play in children, and identified associations should inform community efforts to promote active play in the renovation of recreational facilities.

## Introduction

Physical activity is important for the promotion and maintenance of health (1). Childhood activity, especially play, contributes to healthy emotional, social, and psychological development (2,3) and contributes to healthy physical activity behavior in adulthood (4). Sixty minutes of moderate-to-vigorous physical activity (MVPA) daily is recommended for children and adolescents aged 6 to 17 years (5), but less than half of US children aged 6 to 11 years achieve this target (6). Active play, play that is based on physical activity, is encouraged to achieve these recommendations (7) and to prevent childhood obesity, a stated objective of the American Academy of Pediatrics (3). Childhood obesity is associated with markers of chronic disease, including elevated blood pressure and increased risk of overweight and obesity in adulthood (8).

The physical environment can influence the physical activity behaviors of people (9,10). Public spaces, including playgrounds, provide opportunities for children to interact and engage in physical activity (11,12). Playgrounds are dedicated spaces, alone or in parks, designed for children (often designated by age or height). Playground features include any item in the space such as swings and slides and equipment used for playing as well as items for comfort and aesthetics such as benches, lighting, restrooms, and water fountains. Types of playground features appeal to children and parents, and they affect activity in those spaces (13,14).

One study examined playground features in a national sample of parks and playgrounds and found several elements associated with use and physical activity (15); certain features, such as spinners and splash pads, were associated with increased playground use overall. The study used direct observation and photographs to assess playground features (15).

A recent systematic review highlighted several inconsistent findings across studies that examined environmental features and general park-based physical activity; features such as trails, paths, and lighting were found to be important to park-based physical activity (13). The review also highlighted the need for more studies that use objective measures. Generally, features and conditions of public spaces are thought to be important to the relationship among environments, physical activity, and health outcomes (13,16,17) and important to promote activity, but uncertainty persists about the number and types of amenities necessary to promote the use of public spaces and increase physical activity among visitors (18). These uncertainties apply to playgrounds as well.

A recent article described the development of an audit tool to assess the relationship between features of a playground associated with promoting playground “playability” (the ability of a space to promote active play) and correlations between summary scores on the instrument and park use (19). However, the specific features of a playground space that are important for promoting active play and physical activity to achieve health benefits are still unknown. The objective of our study was to assess associations of playground playability scores with MVPA and energy expenditure and determine whether these associations were independent of other environmental characteristics. We hypothesized that higher scores for playground playability would be associated with greater numbers of children engaged in MVPA and higher energy expenditure.

## Methods

This study was conducted on a sample of 70 audited playgrounds in Chicago, Illinois, that were part of a larger, quasi-experimental study on park renovations (20). We observed 2,712 individuals during the audits. Data collection for characterizing playground features and observation of activity in the playgrounds took place during June and July 2017. Two playgrounds were located in the same park. The research protocol was approved by the University of Illinois at Chicago Office for the Protection of Research Subjects (no. 2016-0497). 

### Measures

#### Playground audits

To evaluate playground features and conditions, we used the Play Space Audit Tool (PSAT), a short audit tool for assessing the playability of playgrounds (19). The tool includes 48 questions about the size, shape, surfaces, paths, vegetation, equipment, rules and regulations, safety, condition of features, and inclusivity of audited playgrounds. Questions are separated into domains for general amenities (15 items, such as benches, restrooms, drinking fountains), surface features (5 items, such as surface hardness, condition), path features (10 items, such as path nearby, width, condition), and play structure features (18 items, such as ≥2 structures, condition). Trained research assistants conducted the playground audits. Reliability of the data was previously reported as good (19).

#### Physical activity

We used the System for Observing Play and Recreation in Communities (SOPARC) tool from June 23, 2017, through July 29, 2017, to assess playground use. SOPARC is widely used, has been demonstrated to be reliable for observation of physical activity in parks, and uses momentary time sampling techniques (21). We used SOPARC in playgrounds on 2 to 6 days, following the protocol established for a related study with 1 or 2 visits to the playground on weekdays and 1 visit on a Saturday for each park (22). Multiple observations were conducted on each visit following the SOPARC protocol (21). We conducted 715 total playground observations, 67% of which were on weekdays and 33% on Saturdays. We used SOPARC to document playground use, and playground users were tallied by sex (male or female), age group (child, teenager, adult, or senior adult), race and ethnicity, activity level (sedentary, moderately active, or vigorously active), and activity type in target areas delineated before the study by trained research staff according to the SOPARC protocol (21). Data on physical activity from SOPARC observations were aggregated across target areas of playgrounds to obtain 1) the total number of individuals in the playground area engaged in MVPA and 2) total energy expenditure (in kcal/kg/min, calculated by multiplying the number of observed sedentary, moderately active, or vigorously active people by 0.051, 0.096, and 0.144 kcal/kg/min, per SOPARC protocol) in the park (21).

### Covariates

To account for the social and economic context in which the playgrounds were located, we obtained data on neighborhood characteristics from 5-year estimates of the American Community Survey (ACS) 2012–2016 and the 2010 US Census (23). We created an index of neighborhood deprivation to describe the socioeconomic status of neighborhoods defined by census tracts in Cook County, Illinois. This index was calculated in principal component analysis as a single-factor representation of several variables at the census tract level. The first principal component represented the linear combination of population factors (percentage of the population that is non-Hispanic White, percentage of adults aged ≤64 years without health insurance, percentage of adults aged ≤64 years receiving Medicaid, percentage of adults aged ≥25 years who graduated from high school, median annual household income, percentage of households below the federal poverty level, percentage of households receiving government financial support, and percentage of households with children headed by unmarried females) that described the greatest proportion of the variability (in this sample, 70.1%). This factor, which served as the index of neighborhood deprivation, ranged from −3.9 to 5.5, with higher values indicating greater deprivation. This index is similar to deprivation indices used nationally (24).

We used a specialized index of disparity, the Index of Concentration at the Extremes (ICE), to assess racial and economic disparity within geographic units (25). We used a variation of this index to assess combined income and racial disparities (ie, social polarization). We used the ACS 5-year estimates (2012–2016) to calculate the difference between the number of non-Hispanic White residents living in households with annual incomes more than $100,000 and the number of Black residents living in households with annual incomes less than $25,000 and dividing by the total population. The ICE ranges from −1 to 1, with values approaching 1 indicating a greater concentration of high-income non-Hispanic White residents and values approaching −1 indicating a greater concentration of low-income Black residents (25).

We obtained crime data from the Chicago Police Department and aggregated these data at the census tract level (26). We calculated crime rates for each crime and each category of crime (violent crime includes homicide, assault, and battery; property crime includes motor vehicle theft, robbery, arson, and burglary) at the census tract level (per 1,000 census tract residents). We summarized the crime data in indices as the first component in a principal components analysis. Indices indicated substantial variability in tract-level crime (74.4% for violent crime and 55.8% for property crime) and were strongly correlated with the overall property and violent crime rates (Pearson correlation coefficients of 0.58 for violent crime rates and 0.98 for property crime rates). We found values for the crime index that ranged from −3.2 to 9.2, with higher values indicating more crime.

We obtained data on hourly temperature, humidity, and precipitation from the National Oceanographic and Atmospheric Administration. We used data on temperature and humidity from the nearest weather station, determined by latitude and longitude, to calculate heat index values for each SOPARC observation.

### Statistical analysis

We used established methods for calculating built environment indices. We calculated playground playability scores for the entire PSAT instrument and for domains of features within the instrument (general amenities, surface, path, and play structure) (27). The scoring process followed 5 basic steps. In Step 1, we coded all 48 features of the audited playground so that higher values indicated a greater likelihood to promote play. In Step 2, we calculated the mean value for each of the 48 features. Step 3 consisted of calculating 5 preliminary scores (for all 48 features and for all features in each of the 4 domains) by adding 1 point to the score for a playground with a feature that had a value greater than or equal to the mean value for that feature in the sample. In Step 4, we calculated mean values of the preliminary scores (overall and in 4 domains) for playgrounds stratified by whether or not the playgrounds had a value for each of the 48 features greater than or equal to the sample mean value for that feature for the preliminary overall score, or whether the playgrounds had a value for each of the domain-specific features greater than or equal to the sample mean for each of the features included in the preliminary domain scores. We calculated the mean difference between the stratified mean preliminary scores for each feature. For example, we calculated the mean preliminary overall score for playgrounds where a drinking fountain was present and for playgrounds where a drinking fountain was not present; we then calculated the difference in those stratified means. Finally, in Step 5, we removed from the final scores features that did not demonstrate internal consistency (contributed to a difference in preliminary scores, between when feature was present or absent, <0.99). The remaining 31 features made up the final score for each park. Details of score development and code to generate these scores are available elsewhere (19).

We compared the characteristics of the SOPARC observations for parks and the census tract–defined neighborhoods containing the parks according to whether the parks had an overall PSAT score at or above the median or below the median. We used χ^2^ and *t* tests to evaluate significance for categorical and continuous variables, respectively.

We calculated incidence rate ratios (IRRs) by using generalized estimating equation negative binomial regression to evaluate the association between the playability score of audited playgrounds and the number of individuals observed engaged in MVPA. This allowed us to accommodate repeated observations of the parks and clustering within census tracts.

Each playability score (overall and domain-specific) was included in models as the primary exposure. In addition to the unadjusted models (Model 1), we also ran several models to adjust for various factors. We adjusted for sex, day of the week, time of day (linear and quadratic), total park area, the ratio of playground to park area, whether the playground was renovated (ie, old playground equipment and ground surfacing were replaced), heat index, and hourly precipitation (Model 2). We then adjusted for Model 2 covariates and neighborhood indices (index of neighborhood deprivation, ICE) and population density (Model 3). Finally, we adjusted for Model 3 covariates and the neighborhood crime index (Model 4).

We used mixed effects models to assess the association between the playability score of audited playgrounds and the total estimated energy expenditure during an observation of the park. We included random intercepts for each playground. Adjustment followed the same scheme described for the negative binomial models.

The study sample included playgrounds that had been recently renovated as part of an evaluation of playground renovations (20). To accommodate this information, we adjusted models for playground renovation status and ran models separately for renovated and unrenovated playgrounds. We also conducted a sensitivity analysis that included only SOPARC observations that observed children (n = 626).

## Results

Thirty-four playgrounds had PSAT scores at or above the median, and 36 playgrounds had scores below the median; the median PSAT score was 18. An average (SD) of 29.0 (35.4) and 10.9 (15.4) individuals were observed in playgrounds with PSAT scores at or above and below the median, respectively (Table 1). On average in parks with PSAT scores at or above the median compared to below the median, we observed more male and female users (16.0 and 13.0 users, respectively, vs 6.3 and 4.6 users, respectively), fewer Black users (35.2 vs 61.7 users, respectively), and more users engaged in moderate physical activity (24.5 vs 15.3 users, respectively). Playgrounds with PSAT scores at or above the median had more activity areas (mean, 25.0) than playgrounds with scores below the median (mean, 20.5) but were less likely to have been renovated (18 vs 29 playgrounds, respectively) (Table 2). Playgrounds with PSAT scores at or above the median were located in neighborhoods with less deprivation, social polarization, and crime.

**Table 1:** Characteristics of Parks During Each Day of System for Observing Play and Recreation in Communities (SOPARC) Observations, by Overall Score (at or Above Median or Below Median) for Play Space Audit Tool (PSAT), Chicago, Illinois, 2017

Variable	PSAT score at or above median (no. of days of observation = 109)^a^	PSAT score below median (no. of days of observation = 97)^a^	*P* value^b^
**Temperature, mean (SD), °F**	79.8 (5.5)	79.7 (4.7)	.83
**Heat index, mean (SD), °F**	82.2 (5.7)	81.7 (4.7)	.46
**Total observed individuals, mean (SD), no.**	29.0 (35.4)	10.9 (15.4)	<.001
**Observed by sex, mean (SD), no.**			
Male	16.0 (20.2)	6.3 (9.2)	<.001
Female	13.0 (16.1)	4.6 (6.7)	<.001
**Observed by race and ethnicity, mean (SD), %**			
Black	35.2 (39.4)	61.7 (45.0)	<.001
Latino	18.8 (27.0)	12.1 (26.3)	.08
White	36.6 (31.8)	20.6 (31.9)	<.001
Other^c^	8.3 (13.0)	5.0 (12.3)	.06
**Observed by age,^d^ mean (SD), %**			
Adult	34.7 (18.8)	30.7 (26.6)	.22
Senior adult	2.0 (4.6)	2.7 (11.1)	.58
Teenager	13.5 (15.3)	18.7 (24.3)	.07
Child	49.7 (19.9)	47.8 (28.0)	.58
**Observed by activity level, mean (SD), %**			
Sedentary	47.1 (17.0)	49.7 (28.5)	.43
Moderate	24.5 (13.2)	15.3 (15.6)	<.001
Vigorous	28.1 (15.2)	34.2 (29.0)	.07
Moderate to vigorous	52.6 (16.9)	49.3 (33.4)	.36
**Average scan start time, mean (SD)**	2:51 pm (2:00 h:min)	3:14 pm (2:15 h:min)	.50
**Day of observation, n (%)**			
Monday	11 (10.1)	7 (7.2)	.88
Thursday	26 (23.8)	26 (26.8)	
Friday	36 (33.0)	32 (33.0)	
Saturday	36 (33.0)	32 (33.0)	

a The PSAT is used to assess the playability (the ability of a space to promote active play) of playgrounds. Median PSAT score for this sample was 18 and ranged from 9 to 26.

b *P* values were determined by χ^2^ tests (categorical variables) or *t* tests (continuous variables).

c Includes individuals determined by the trained researchers not to be Black, White, or Latino. This categorization is subject to the limitations of visually determining race and ethnicity and will include non-Hispanic Asian, Pacific Islander, and Native American individuals.

d Per SOPARC protocol, child defined as aged 0–12 years; teenager, 13–20 years; adult, 21–59 years; senior, ≥60 years. No ages were verified for this study.

**Table 2:** Park and Neighborhood Characteristics for Playgrounds Audited With the Play Space Audit Tool (PSAT), by Overall PSAT Score (at or Above Median or Below Median), Chicago, Illinois, 2017

Characteristics	PSAT score at or above median^a^ (n = 33)	PSAT score below median^a^ (n = 36)	*P* value^b^
**Park**			
Park acreage, median (IQR)	1.8 (0.4–9.8)	0.7 (0.2–1.6)	.07
Playground acreage, median (IQR)	0.1 (0.1–0.2)	0.1 (0.1–0.2)	.09
Irregular playground shape, no. (%)	31 (96.9)	31 (86.1)	.28
Posted opening time, median (IQR)	6 am (6 am–6 am)	6 am (6 am–6 am)	>.99
Posted closing time, median (IQR)	11 pm (9 pm–11 pm)	11 pm (9 pm–11 pm)	>.99
Playground signage indicated appropriate age group, n (%)			
2–5 y	8 (24.2)	14 (38.9)	.19
5–12 y	1 (3.0)	0	.47
No. of activity areas, median (IQR)	25.0 (21.0–45.0)	20.5 (15.0–26.5)	.007
Renovated playground, no. (%)	18 (54.5)	29 (80.6)	.04
**Neighborhood measures, mean (SD)**			
Population density per square mile	17,034 (8,866)	13,708 (6,741)	.09
Non-Hispanic White, %	54.6 (33.5)	29.5 (33.2)	.003
Adults aged ≤64 years without health insurance, %	16.4 (10.9)	21.1 (10.6)	.07
Adults aged ≤64 years receiving Medicaid, %	13.0 (10.6)	23.4 (14.0)	.001
Adults aged ≥25 years who graduated from high school, %	86.2 (11.8)	82.7 (9.5)	.29
Median annual household income, $	54,158 (28,758)	39,801 (26,749)	.04
Households below federal poverty level, %	15.8 (11.6)	26.7 (15.2)	.001
Households receiving government financial support, %	33.3 (24.4)	55.0 (27.5)	.001
Households with children headed by unmarried females, %	35.6 (27.4)	53.0 (27.2)	.01
Households receiving SNAP, %	18.5 (14.3)	32.5 (18.3)	<.001
Households receiving TANF, %	3.2 (2.3)	5.9 (3.5)	<.001
Violent crime rate per 1,000 residents^c^	21.1 (17.3)	45.1 (30.7)	<.001
Property crime rate per 1,000 residents^c^	12.5 (6.8)	22.4 (13.8)	<.001
Neighborhood indices, mean (SD)			
Index of neighborhood deprivation^d^	0.04 (2.33)	2.08 (2.60)	.001
ICE^e^	−0.05 (0.30)	−0.28 (0.31)	.003
Crime index^f^	−0.76 (2.27)	1.50 (3.60)	.003
PSAT domain scores, mean (SD)^a^			
General amenities	7.8 (1.4)	5.0 (1.6)	<.001
Surface	1.8 (0.5)	1.4 (0.6)	.003
Path	5.0 (0.9)	4.0 (2.1)	.02
Play structure	9.2 (1.1)	6.6 (1.6)	<.001

Abbreviations: ICE, Index of Concentration at the Extremes; IQR, interquartile range; SNAP, Supplemental Nutrition Assistance Program; TANF, Temporary Assistance for Needy Families.

a The PSAT is used to assess the playability (the ability of a space to promote active play) of playgrounds. Maximum possible score is 31, and minimum possible score is 0. Median PSAT score for this sample was 18 (range, 9–26).

b *P* values were determined by χ^2^ tests (categorical values) or *t* tests (continuous variables).

c Violent (homicide, assault, battery) and property (motor vehicle theft, robbery, arson, burglary) crime rates (per 1,000 residents) were calculated for census tracts.

d Index of neighborhood deprivation is the first principal component of census tract–level percentage of non-Hispanic White residents, percentage of adults aged ≤64 years without health insurance, percentage of adults aged ≤64 years receiving Medicaid, percentage of adults aged ≥25 years who have graduated from high school, median annual household income, percentage of households below the federal poverty level, percentage of households receiving government financial support, and percentage of households with children headed by unmarried females. Higher values indicate less deprivation.

e A modified version of ICE was used to measure spatial social polarization at the census tract level. It was calculated as the difference between the number of non-Hispanic White residents living in households with annual incomes >$100,000 and the number of Black residents living in households with annual incomes <$25,000 and dividing by the total population. ICE ranges from −1 to 1, with values approaching 1 indicating a greater concentration of high-income non-Hispanic White residents and values approaching −1 indicating a greater concentration of low-income Black residents (25).

f Crime data obtained from Chicago Police Department and aggregated at the census tract level (26).

Negative binomial models for the number of individuals observed engaged in MVPA found significant associations in unadjusted models for overall PSAT scores and PSAT scores for general amenities and play structures, with higher playability scores associated with more individuals observed engaging in MVPA (Table 3). Associations in all parks were robust to adjustment, with 1-point higher general amenities and play structure scores associated with 28% (IRR, 1.28; 95% CI, 1.08–1.52) and 15% (IRR, 1.15, 95% CI, 1.00–1.31) more individuals engaging in MVPA, respectively. We observed no associations of playability scores with MVPA in adjusted models for unrenovated playgrounds. In fully adjusted models for renovated playgrounds, 1-point higher general amenities and play structure scores were associated with 31% (IRR, 1.31; 95% CI, 1.10–1.57) and 22% (IRR, 1.22; 95% CI, 1.03–1.43) more individuals engaged in MVPA, respectively.

**Table 3:** Incidence Rate Ratios^a^ for Number of Additional Individuals in Park Engaged in Moderate-to-Vigorous Physical Activity in SOPARC Scans per 1-Point Increase in PSAT Scores, Overall and by PSAT Domain, Chicago, Illinois, 2017

PSAT score domain^b^	Incidence rate ratio (95% CI)			
	Model 1	Model 2	Model 3	Model 4
**All parks (N = 715)**				
Overall	1.10 (1.01–1.19)^c^	1.07 (1.01–1.14)^c^	1.07 (1.00–1.15)^c^	1.04 (0.99–1.10)
General amenities	1.34 (1.20–1.49)^c^	1.25 (1.12–1.39)^c^	1.25 (1.12–1.40)^c^	1.28 (1.08–1.52)^c^
Surface	0.93 (0.55–1.58)	0.98 (0.66–1.45)	0.93 (0.59–1.45)	0.86 (0.66–1.13)
Path	0.96 (0.80–1.16)	0.88 (0.79–0.99)	0.91 (0.77–1.06)	0.97 (0.88–1.07)
Play structure	1.20 (1.06–1.36)^c^	1.22 (1.09–1.36)^c^	1.20 (1.07–1.35)^c^	1.15 (1.00–1.31)^c^
**Parks with unrenovated playgrounds (n = 250)^d^**				
Overall	1.02 (0.89–1.16)	1.07 (0.96–1.08)	1.00 (0.94–1.07)	1.01 (0.92–1.12)
General amenities	1.23 (1.05–1.44) **c**	1.13 (0.99–1.28)	1.13 (0.98–1.30)	1.04 (0.84–1.30)
Surface	0.80 (0.47–1.38)	1.03 (0.75–1.42)	0.92 (0.61–1.38)	0.93 (0.69–1.23)
Path	0.99 (0.77–1.27)	0.87 (0.66–1.15)	0.85 (0.64–1.13)	1.01 (0.77–1.33)
Play structure	0.96 (0.72–1.27)	1.10 (0.93–1.30)	1.07 (0.87–1.32)	1.08 (0.86–1.34)
**Parks with renovated playgrounds (n = 465)^d^**				
Overall	1.17 (1.09–1.24)^c^	1.07 (1.04–1.21)^c^	1.13 (1.05–1.22)^c^	1.09 (0.99–1.19)
General amenities	1.37 (1.21–1.55)^c^	1.30 (1.11–1.52)^c^	1.33 (1.18–1.49)^c^	1.31 (1.10–1.57)^c^
Surface	0.97 (0.36–2.62)	1.38 (0.51–3.72)	0.96 (0.41–2.24)	1.03 (0.63–1.67)
Path	0.91 (0.74–1.11)	0.85 (0.76–0.97)	0.87 (0.71–1.06)	0.92 (0.84–1.02)
Play structure	1.29 (1.12–1.48)^c^	1.30 (1.11–1.52)^c^	1.29 (1.01–1.63)^c^	1.22 (1.03–1.43)^c^

Abbreviations: PSAT, Play Space Audit Tool; SOPARC, System for Observing Play and Recreation in Communities.

a Incidence rate ratios were obtained from negative binomial generalized estimating equation models. Model 1 is unadjusted. Model 2 is adjusted for sex, day of week, time of day, time of day squared, park area, the ratio of playground to park area, renovation, heat index, and hourly precipitation. Model 3 is adjusted for all Model 2 covariates and the following census tract–defined variables: neighborhood deprivation index, the index of concentration at the extremes, and population density. Model 4 is adjusted for all Model 3 covariates and a crime index for the census tract.

b The PSAT is used to assess the playability (the ability of a space to promote active play) of playgrounds. Domain-specific scores ranged from 9 to 26 (overall), 2 to 10 (general amenities), 0 to 1 (surface), 0 to 6 (path), and 0 to 11 (play structure).

c *P* < .05; determined by robust standard error estimates.

d Models stratified by whether the audited playground had been renovated or was unrenovated were not adjusted for renovation status.

Mixed models for energy expenditure identified significant associations for overall score and scores for general amenities and play structures, with higher scores for playability associated with greater energy expenditure among observed individuals (Table 4). In unadjusted models for all parks, 1-point higher overall and general amenities scores were associated with a 0.13 (95% CI, 0.02 to 0.23) and 0.41 (95% CI, 0.21 to 0.61) kcal/kg/min greater energy expenditure, respectively. In fully adjusted models for all parks, a 1-point higher general amenities score was associated with a 0.42 (95% CI, 0.15 to 0.68) kcal/kg/min greater energy expenditure. We observed no associations among unrenovated playgrounds. In unadjusted models for renovated playgrounds, 1-point higher overall scores and scores for general amenities and play structures were associated with 0.17 (95% CI, 0.05 to 0.29), 0.44 (95% CI, 0.22 to 0.66), and 0.29 (95% CI, 0.03 to 0.55) kcal/kg/min greater energy expenditure, respectively. The magnitudes of the associations were not attenuated by adjustment, although the associations for the overall and play structure scores were no longer significant. In fully adjusted models for renovated playgrounds, a 1-point general amenities score was associated with 0.51 (95% CI, 0.24 to 0.79) kcal/kg/min greater energy expenditure.

**Table 4:** Estimated Energy Expenditure^a^ of Individuals Observed in SOPARC Scans per 1-Point Increase in Scores, Overall and PSAT Domain, Chicago, Illinois, 2017

PSAT score domain^b^	β (95% CI)			
	Model 1	Model 2	Model 3	Model 4
**All parks (N = 715)**				
Overall	0.13 (0.02 to 0.23)^c^	0.09 (−0.02 to 0.19)	0.08 (−0.04 to 0.20)	0.08 (−0.04 to 0.20)
General amenities	0.41 (0.21 to 0.61)^c^	0.31 (0.11 to 0.52)^c^	0.40 (0.15 to 0.65)^c^	0.42 (0.15 to 0.68)^c^
Surface	−0.07 (−0.83 to 0.69)	0.02 (−0.70 to 0.73)	−0.02 (−0.75 to 0.71)	−0.02 (−0.75 to 0.72)
Path	−0.07 (−0.34 to 0.19)	−0.19 (−0.45 to 0.06)	−0.18 (−0.44 to 0.08)	−0.18 (−0.44 to 0.08)
Play structure	0.22 (−0.02 to 0.45)	0.21 (−0.02 to 0.44)	0.18 (−0.07 to 0.43)	0.18 (−0.07 to 0.44)
**Parks with unrenovated playgrounds (n = 250)^d^**				
Overall	0.03 (−0.21 to 0.26)	0 (−0.21 to 0.20)	0.15 (−0.08 to 0.37)	0.09 (−0.16 to 0.35)
General amenities	0.32 (−0.14 to 0.77)	0.02 (−0.42 to 0.46)	0.31 (−0.20 to 0.82)	0.13 (−0.58 to 0.83)
Surface	−0.36 (−1.71 to 0.98)	0 (−1.23 to 1.22)	0.28 (−0.99 to 1.55)	0.22 (−1.03 to 1.46)
Path	−0.06 (−0.61 to 0.49)	−0.01 (−0.55 to 0.54)	0.25 (−0.36 to 0.86)	0.24 (−0.36 to 0.83)
Play structure	−0.10 (−0.69 to 0.49)	−0.02 (−0.54 to 0.50)	0.29 (−0.28 to 0.86)	0.19 (−0.40 to 0.78)
**Parks with renovated playgrounds (n = 465)^d^**				
Overall	0.17 (0.05 to 0.29)^c^	0.15 (0.03 to 0.28)^c^	0.14 (0 to 0.29)	0.14 (−0.01 to 0.29)
General amenities	0.44 (0.22 to 0.66)^c^	0.41 (0.19 to 0.64)^c^	0.50 (0.23 to 0.77)^c^	0.51 (0.24 to 0.79)^c^
Surface	0.04 (−0.89 to 0.98)	0.20 (−0.71 to 1.11)	−0.06 (−1.02 to 0.89)	−0.12 (−1.08 to 0.85)
Path	−0.11 (−0.41 to 0.19)	−0.19 (−0.48 to 0.10)	−0.18 (−0.47 to 0.12)	−0.18 (−0.47 to 0.12)
Play structure	0.29 (0.03 to 0.55)^c^	0.31 (0.05 to 0.57)^c^	0.27 (−0.02 to 0.55)	0.26 (−0.04 to 0.55)

Abbreviations: PSAT, Play Space Audit Tool; SOPARC, System for Observing Play and Recreation in Communities.

a Energy expenditure estimates, in kcal/kg/min, were obtained from mixed effects regression models. Model 1 is unadjusted. Model 2 is adjusted for sex, day of week, time of day, time of day squared, park area, the ratio of playground to park area, renovation, heat index, and hourly precipitation. Model 3 is adjusted for all Model 2 covariates and the following census tract–defined variables: neighborhood deprivation index, the index of concentration at the extremes, and population density. Model 4 is adjusted for all Model 3 covariates and a crime index for the census tract.

b The PSAT is used to assess the playability (the ability of a space to promote active play) of playgrounds.

c *P* < .05; determined by *F* test.

d Models stratified by whether the audited playground had been renovated or was unrenovated were not adjusted for renovation status.

The sensitivity analysis of only SOPARC scans with observed children generated results that were nearly identical to the main analysis (Supplemental Table 1 and Supplemental Table 2 in Appendix). Because of the small number of unrenovated playgrounds in the sensitivity analyses, we present overall and renovated playground results only.

## Discussion

Playground playability as measured by the PSAT was significantly associated with greater MVPA for the general amenities and play structure domains in all playgrounds and in renovated playgrounds. Associations were observed between the overall PSAT score for the entire instrument and greater MVPA in all playgrounds and in renovated playgrounds, although these associations were no longer significant after adjustment for neighborhood crime. We observed significant associations between overall and general amenities scores and greater energy expenditure in unadjusted models for all playgrounds and for renovated playgrounds. In fully adjusted models for all playgrounds, only the general amenities score was associated with significantly greater energy expenditure. In minimally adjusted regression models (Model 2) the overall, general amenities, and play structure scores were associated with greater energy expenditure in renovated playgrounds, but after adjustment, only the overall and play structure scores remained significant. No significant associations were observed between scores and energy expenditure in unrenovated playgrounds, suggesting that park playgrounds with a diverse mix of play features that are more likely to be in good condition are associated with higher levels of physical activity.

Numerous studies have explored the relationship between playground features and physical activity, but most have been conducted in school playgrounds rather than public parks. Prior research on playground renovations reported mixed results; some showed greater physical activity and less sedentary time (28,29), and a more recent study showed mixed results that depended on neighborhood income level (22). A study that examined playground characteristics on elementary school grounds in Denver, Colorado, found significant associations between increased density of features and use among all children and between density of features and MVPA among girls but not boys (30). A study that assessed playgrounds by using the Environmental Assessment of Public Recreation Spaces (EAPRS) tool found MVPA and overall use were higher in playgrounds that had more varied play facilities and had fewer natural design elements or plantings (31). Our study evaluated summary scores by domains of features and, thus, is not directly comparable to much of the prior literature, but the findings that higher overall scores and scores for general amenities and play structure were associated with more individuals engaged in MVPA is in concordance with numerous previous reports (30,31).

A national study of parks in 2016 found that each additional playground structure was associated with more physical activity (15). Specifically, spinning structures and splashpads were associated with increased use and MVPA. Our study similarly found the importance of play features associated with MVPA and higher energy expenditure, particularly in renovated playgrounds.

We observed no associations between scores for path and surface features and either MVPA or energy expenditure. This null finding in the present study aligns with the associations reported in the previously mentioned national study (15). The absence of association may accurately represent an absence of association between playground surface and path features and park activity or reflect characteristics of the sample of playgrounds. Most playgrounds audited were located in urban playlots, and paths may not influence the number of individuals engaged in or the intensity of the physical activity in a space for small parks. Additionally, we found relatively little variability in surface features in our sample. Associations between surface score and physical activity might be observed in a sample of playgrounds that is more diverse than ours in the presence and condition of surface features.

Associations between play space scores and MVPA were observed in fully adjusted models for general amenities and play structure scores in all playgrounds and renovated playgrounds. The association of general amenities and play structure scores with MVPA was not found in unrenovated playgrounds. The reasons for these differences in unrenovated playgrounds are unknown. A lack of variability in general amenities in unrenovated playgrounds may have contributed to the absence of association with MVPA.

That unrenovated playgrounds had higher PSAT scores than renovated playgrounds was unexpected. However, this finding is consistent with the parent study, which found that MVPA and use of renovated playgrounds declined over time across neighborhood demographics (22). The study authors suggested that the renovations may not have met the needs of residents in low-income and predominantly Black neighborhoods, which increased disparities in playground use across neighborhoods and highlighted the importance of involving community members in neighborhood-level improvement efforts.

Play space features could influence physical activity by numerous pathways. Attractive playgrounds with multiple features in good condition will appeal to guardians and children, encouraging greater use. Activity panels, cluster points, and nooks encourage the congregation of children, enhancing the social appeal of the playground, which may lead to greater use (32). The types of features present might elicit different intensities of exertion. Some features may encourage vigorous activity (swinging, climbing), while other features might demand lower-intensity activity (31).

### Strengths and limitations

Our study has several strengths. In addition to use of a brief, reliable, simple audit instrument, trained research assistants collected data on playground features and observed activity according to standardized methods (19,21). The scoring system used summarized playground features in domains that maintain the maximum variability of features while maintaining the internal consistency of each domain’s score and demonstrated consistency with playground and neighborhood characteristics. The scoring system facilitated comparison of playgrounds within the sample and may be a more consistent method than others for evaluating playground features in relation to MVPA and energy expenditure. Additionally, we were able to make observations on playgrounds that had undergone renovations, and we included a wide variety of data to characterize the neighborhoods where the playgrounds were located.

Our study also had several limitations. The SOPARC observations of playground activity rely on trained observers who assess the age and race of observed individuals, and data collection methods preclude delineation of physical activity by age when individuals of multiple ages and physical activity levels are observed in the same target area during a single observation. The PSAT is limited in the number of items assessed to maintain a simple-to-use format, and although it is designed to capture data on major aspects of play and determinants of use, it may lack items that could be important. For example, at least 1 study found that splashpads were important to overall use and MVPA (15). Although the PSAT has a write-in space to capture any item not otherwise assessed in the instrument, it does not have a specific item for splashpads. However, this was not a problem in our study because none of the playgrounds had splashpads. The scores described are sample-dependent, and variables included in scores depend on the joint distribution of features within a study sample. The instrument has not been tested in nonurban areas and may not capture data on determinants of use, MVPA, and energy expenditure in less populated areas. The ability to make causal inferences between playability scores and MVPA and energy expenditure is limited because the observations were cross-sectional.

### Conclusion

Playground features were significantly associated with MVPA and energy expenditure. These associations were robust to adjustment for individual, environmental, and neighborhood factors, and they support previous findings indicating that the features of public spaces are important for physical activity. The results of our study suggest that greater number and quality of features relative to other playgrounds is associated with greater physical activity among children. These findings are relevant for numerous community groups. Managed parks and recreation departments and programs have a vital role in influencing the health of children (33), and municipalities and educational organizations such as schools can play an important role in promoting the health of the children of their communities. Our study provides evidence that can support these community groups when they advocate for playgrounds that encourage children to be physically active while playing (34,35). Communities should advocate for and design playgrounds that encourage active play.

## Appendix. Supplemental Tables

**Table:** Appendix. Supplemental Table 1. Results of Sensitivity Analysis of Park Scans Containing Children in Parks: Incidence Rate Ratios^a^ for Number of Additional Individuals in Park Engaged in Moderate-To-Vigorous Physical Activity in SOPARC Scans per 1-Point Increase in PSAT Scores, Overall and by PSAT Domain, Chicago, Illinois, 2017

PSAT score domain^b^	Incidence rate ratio (95% CI)			
	Model 1	Model 2	Model 3	Model 4
**All parks (N = 626)**				
Overall	1.09 (1.01–1.17)^c^	1.10 (1.03–1.18)^c^	1.10 (1.02–1.19)^c^	1.10 (1.02–1.19)^c^
General amenities	1.28 (1.16–1.42)^c^	1.16 (1.03–1.30)^c^	1.17 (1.00–1.37)^c^	1.19 (1.01–1.40)^c^
Surface	0.95 (0.58–1.57)	1.36 (0.95–1.93)	1.59 (1.06–2.38)^c^	1.92 (1.19–3.10)^c^
Path	0.97 (0.81–1.15)	0.97 (0.84–1.11)	1.01 (0.87–1.17)	1.01 (0.87–1.18)
Play structure	1.18 (1.05–1.33)^c^	1.29 (1.15–1.45)^c^	1.28 (1.11–1.47)^c^	1.31 (1.13–1.51)^c^
**Parks with renovated playgrounds (n = 406)^d^**				
Overall	1.16 (1.09–1.23)^c^	1.17 (1.09–1.27)^c^	1.16 (1.06–1.28)^c^	1.14 (1.02–1.27)^c^
General amenities	1.34 (1.19–1.51)^c^	1.18 (1.03–1.37)^c^	1.16 (0.96–1.39)	1.24 (1.07–1.45)^c^
Surface	0.98 (0.38–2.53)	1.77 (0.94–3.35)	1.70 (0.83–3.51)	2.45 (1.45–4.14)^c^
Path	0.91 (0.75–1.11)	0.96 (0.80–1.15)	0.95 (0.81–1.10)	0.89 (0.77–1.03)
Play structure	1.28 (1.13–1.46)^c^	1.47 (1.32–1.64)^c^	1.46 (1.27–1.66)^c^	1.42 (1.15–1.76)^c^

Abbreviations: PSAT, Play Space Audit Tool; SOPARC, System for Observing Play and Recreation in Communities.

^a^ Incidence rate ratios were obtained from negative binomial generalized estimating equation models. Model 1 is unadjusted. Model 2 is adjusted for sex, day of week, time of day, time of day squared, park area, the ratio of playground to park area, renovation, heat index, and hourly precipitation. Model 3 is adjusted for all Model 2 covariates and the following census tract–defined variables: neighborhood deprivation index, the index of concentration at the extremes, and population density. Model 4 is adjusted for all Model 3 covariates and a crime index for the census tract.

^b^ The PSAT is used to assess the playability (the ability of a space to promote active play) of playgrounds.

^c^ *P* < .05; determined by robust standard error estimates.

^d^ Models were run stratified by renovation status. Because of a small number of observations for unrenovated playgrounds and problems with convergence of the statistical estimation algorithms in model fitting, we excluded unrenovated playgrounds from the stratified analysis.

**Table:** Appendix. Supplemental Table 2. Results of Sensitivity Analysis of Park Scans Containing Children in Parks: Estimated Energy Expenditure^a^ of Individuals Observed in SOPARC Scans per 1-Point Increase in Scores, Overall and PSAT Domain, Chicago, Illinois, 2017

PSAT score domain^b^	β (95% CI)			
	Model 1	Model 2	Model 3	Model 4
**All parks (N = 626)**				
Overall	0.12 (0.02 to 0.23)^c^	0.13 (−0.15 to 0.42)	0.21 (0.09 to 0.51)	0.21 (−0.09 to 0.51)
General amenities	0.39 (0.18 to 0.60)^c^	0.25 (−0.33 to 0.84)	0.39 (−0.24 to 1.03)	0.34 (−0.32 to 1.00)
Surface	−0.04 (−0.81 to 0.73)	0.64 (−1.19 to 2.48)	1.62 (−0.39 to 3.63)	1.52 (−1.00 to 4.03)
Path	−0.08 (−0.35 to 0.19)	−0.25 (−0.97 to 0.47)	−0.10 (−0.86 to 0.67)	−0.06 (−0.83 to 0.71)
Play structure	0.21 (−0.03 to 0.45)	0.45 (−0.13 to 1.03)	0.46 (−0.15 to 1.06)	0.58 (−0.03 to 1.19)
**Parks with renovated playgrounds (n = 406)^d^**				
Overall	0.17 (0.05 to 0.29)^c^	0.24 (−0.06 to 0.53)^c^	0.32 (0.04 to 0.60)^c^	0.36 (0.02 to 0.69)^c^
General amenities	0.44 (0.22 to 0.67)	0.29 (−0.29 to 0.87)	0.39 (−0.17 to 0.94)	0.40 (−0.19 to 0.99)
Surface	0.06 (−0.90 to 1.01)	1.05 (−1.38 to 3.47)	2.29 (−0.30 to 4.87)	2.46 (−0.18 to 5.10)
Path	−0.12 (−0.43 to 0.19)	−0.34 (−1.18 to 0.50)	−0.16 (−0.98 to 0.66)	−0.21 (−1.09 to 0.68)
Play structure	0.31 (0.04 to 0.57)^c^	0.59 (0.13 to 1.06)^c^	0.59 (0.13 to 1.04)^c^	0.75 (0.18 to 1.32)^c^

Abbreviations: PSAT, Play Space Audit Tool; SOPARC, System for Observing Play and Recreation in Communities.

^a^ Energy expenditure estimates, in kcal/kg/min, were obtained from mixed effects regression models. Model 1 is unadjusted. Model 2 is adjusted for sex, day of week, time of day, time of day squared, park area, the ratio of playground to park area, renovation, heat index, and hourly precipitation. Model 3 is adjusted for all Model 2 covariates and the following census tract–defined variables: neighborhood deprivation index, the index of concentration at the extremes, and population density. Model 4 is adjusted for all Model 3 covariates and a crime index for the census tract.

^b^ The PSAT is used to assess the playability (the ability of a space to promote active play) of playgrounds.

^c^ *P* < .05; determined by *F* test.

^d^ Models were run stratified by renovation status. Because of a small number of observations for unrenovated playgrounds and problems with convergence of the statistical estimation algorithms in model fitting, we excluded unrenovated playgrounds from the stratified analysis.
